# Ketogenic diet therapy in adolescents and adults with epilepsy: A scoping review of cognitive outcomes

**DOI:** 10.1002/epi4.70379

**Published:** 2026-11-14

**Authors:** Tabarek Ibrahim, Anna Miley Åkerstedt, Ann‐Sofie Rudberg

**Affiliations:** ^1^ Study Programme in Psychology Karolinska Institutet Stockholm Sweden; ^2^ Department of Clinical Neuroscience Karolinska Institutet Stockholm Sweden; ^3^ Women's Health and Allied Health Professionals Theme Karolinska University Hospital Stockholm Sweden; ^4^ Department of Clinical Sciences, Danderyd Hospital Karolinska Institutet Stockholm Sweden; ^5^ Department of Neurology Danderyd Hospital Stockholm Sweden

**Keywords:** adolescents, adults, cognition, dietary therapy, seizure disorder

## Abstract

**Objective:**

The ketogenic diet treatment (KDT) is an established non‐pharmacological treatment for drug‐resistant epilepsy (DRE). However, rigorous research for seizure efficacy based on randomized controlled studies in adolescents and adults remains limited, and cognitive outcomes are rarely assessed using standardized measures.

**Methods:**

This scoping review has mapped evidence on cognitive outcomes of KDT in adolescents and adults with epilepsy. Following PRISMA‐ScR guidelines, PubMed and Embase were searched for studies published between 1986 and 2026. Eligibility for this review required inclusion of adolescents or adults with epilepsy receiving KDT and reporting cognitive outcomes.

**Results:**

Of 951 records, 19 studies were included. Overall, KDT was associated with favorable seizure and cognitive outcomes, although reports of cognitive outcomes were generally subjective.

**Significance:**

Rigorous research designs were scarce, and evidence remains limited; future studies should use stronger designs, larger samples, standardized cognitive assessments, and longer follow‐up periods.

**Plain Language Summary:**

Research shows that people with epilepsy often experience difficulties with the brain's ability to process information. While dietary treatments have been shown to be effective for seizure control, less is known about their effects on cognition. We reviewed published research examining cognitive outcomes in adolescents and adults with epilepsy following dietary treatment. Most studies suggested that these diets had a positive effect on aspects of cognitive functioning. However, the available evidence remains limited. More studies with better designs using well‐established cognitive assessments are needed to better understand these effects.

**Key points**

- This scoping review examined KDT as a treatment for adolescents and adults with epilepsy and its effect on cognition.
- Most included studies reported positive effects on cognition following KDT.
- The results were limited by heterogeneity, subjective assessments, and limited methodological rigor.
- Current research is insufficient to draw firm conclusions about the effects of KDT on cognition in adolescents and adults with epilepsy.
- Future research should include controlled studies using standardized cognitive measures.

Epilepsy is a chronic neurological disorder that can develop at any age and affects more than 50 million people worldwide.^1^ Approximately one third of people with epilepsy have drug‐resistant epilepsy (DRE), which is associated with higher morbidity and mortality. Although surgery and vagus nerve stimulation may benefit selected patients, they are not suitable or sufficient for all individuals with DRE.^2^, ^3^ These limitations underscore the need for complementary non‐pharmacological approaches that influence seizure susceptibility and treatment response alongside antiseizure medications (ASMs). Dietary therapies are non‐pharmacological treatment options for DRE, particularly in pediatric populations. Ketogenic diet (KD) and modified Atkins diet (MAD) are two forms of ketogenic diet therapy (KDT) commonly used in the dietary treatment of epilepsy.^4^, ^5^ KD is a high‐fat, low‐carbohydrate dietary therapy that induces ketosis, whereas MAD is a less restrictive ketogenic alternative.^5^, ^6^ Although ketosis is thought to support neuronal function, the mechanisms underlying the anticonvulsant effects of KDT remain incompletely understood.

Due to strong evidence in children, dietary therapies have become established treatments for pediatric epilepsy. Systematic reviews report improvements in seizure control^4^ and occasional subjective cognitive benefits.^7^, ^8^, ^9^ Rigorous research beyond childhood remains limited, although studies in adults have recently emerged, including research on cognitive outcomes.^8^, ^10^

Cognitive difficulties are common in epilepsy, with up to 50% of individuals demonstrating clinical cognitive impairments on standardized tests or reporting memory difficulties that significantly affect everyday life.^11^ Cognition encompasses domains such as memory, attention, executive functioning, processing speed, and language, which can be assessed using standardized neuropsychological tests (^12^). These cognitive difficulties represent an important component in the disease burden for patients with epilepsy. While seizures are episodic, cognitive difficulties may act as a constant daily reminder of the condition and interfere with everyday functioning.^11^, ^13^ These difficulties are likely due to the interaction of multiple factors, including high seizure frequency, brain lesions, and the side effects of pharmacological treatments. Research on how different treatment approaches influence cognitive functioning is therefore important. Research is particularly limited in adult populations, in which very few studies compare the cognitive outcomes of dietary therapy in epilepsy to those of pediatric patients.^8^ Several studies suggest that KDT may influence cognitive outcomes. A systematic overview reported possible improvements in alertness, potentially linked to reduced seizure activity and effects of ketone bodies.^9^ However, consensus remains limited, and objective evidence is scarce, particularly for adolescents and adults.

Dietary therapy studies rarely assess cognition using objective, standardized measures.^8^, ^9^ Outcomes are often reported subjectively by parents, patients, carers, or clinicians. Given the limited and heterogeneous research, a scoping review is long overdue. This scoping review maps the current evidence of cognitive outcomes associated with dietary therapies in adults and adolescents with epilepsy, and it identifies reported cognitive outcomes, assessment methods, and knowledge.

## 1 METHOD

This scoping review maps the evidence of cognitive outcomes associated with KDT in adolescent and adult epilepsy populations. It follows PRISMA‐ScR guidance to identify available evidence and knowledge gaps.^14^

### 1.1 Protocol and registration

No protocol was registered for this scoping review.

### 1.2 Eligibility criteria

Eligibility was defined using the PICO framework: adolescents or adults with epilepsy, KDT as the intervention, with or without a comparison group, and reported cognitive outcomes. Cognitive outcomes included objectively assessed cognitive performances as well as subjectively reported cognitive changes. Exclusion criteria included non‐epilepsy primary conditions, such as glucose transporter type 1; studies in which KDT was not the primary intervention and diet‐specific results could not be extracted; multimodal interventions or a large number of confounders where KDT effects could not be isolated; mixed‐age populations where age‐specific results for participants aged ≥10 years could not be extracted; exclusively pediatric populations; and articles not written in English. PubMed and Embase were selected for their broad medical and neuropsychological coverage. Adolescence was defined according to the World Health Organization criteria (10–19 years).^15^ The search covered publications from 1986 to 2026 in PubMed and 1995 to 2026 in Embase, based on the earliest records available in each database.

### 1.3 Information source/database

Literature searches were conducted in the electronic databases PubMed and Embase. The search was based on the earliest records available in each database, covering the period 1986–2026 in PubMed and 1995–2026 in Embase.

### 1.4 Search strategy

The initial search strategy was intentionally broad and included constructs commonly investigated in relation to epilepsy, including cognition and sleep. During preliminary screening of the literature, it became apparent that sleep belonged to a distinct research domain and was therefore not included as part of the PICO criteria. The focus of the review was limited to cognitive outcomes, but the original search was retained to ensure comprehensive coverage of the available literature. Search terms included “epilepsy”, “seizure disorder”, “ketogenic diet”, “Atkins diet”, “cognition”, and “sleep”. Where available, database‐specific indexing terms and Medical Subject Headings (MeSH) were used. Boolean operators (AND/OR) were used in combination to broaden or narrow the search as necessary. The full search strategies and search blocks for each database are presented in Tables 1 and 2.

**TABLE 1:** PubMed Database Search Strategy and Results.

Database: PubMed	Keywords	Limitations (filter, limits, refine)	Number of hits
#1	“diet, ketogenic”[MeSH Terms] OR “ketogenic diet*”[Title/Abstract] OR “atkins diet*”[Title/Abstract]		6515
#2	“epilepsy”[MeSH Terms] OR “seizure disorder*”[Title/Abstract] OR “epilep*”[Title/Abstract]		215 982
#3	“cognition”[MeSH Terms] OR “sleep”[MeSH Terms] OR “cognit*”[Title/Abstract] OR “sleep*”[Title/Abstract]		1 027 265
#4	#1 AND #2 AND #3		395

**TABLE 2:** Embase Database Search Strategy and Results.

Database: Embase	Keywords	Limitations (filter, limits, refine)	Number of hits
#1	‘ketogenic diet’/exp		11 571
#2	‘atkins diet’/exp		672
#3	‘ketogenic diet*’:ab,ti,kw OR ‘atkins diet*’:ab,ti,kw		9513
#4	#1 OR #2 OR #3		12 955
#5	‘epilepsy’/exp		345 343
#6	epilep*:ab,kw,ti OR ‘seizure disorder*’:ab,kw,ti		287 770
#7	#5 OR #6		395 743
#8	‘cognition’/exp		3 804 058
#9	‘sleep’/exp		368 301
#10	cognit*:ab,kw,ti OR sleep*:ab,kw,ti		1 345 238
#11	#8 OR #9 OR #10		4 552 943
#12	#4 AND #7 AND #11		1759
#13	#12	AND (‘adolescent’:ag OR ‘adult’:ag)	556

### 1.5 Selection of sources of evidence

Study selection was conducted in two stages. In the first stage, the literature was screened based on titles and abstracts using the inclusion criteria of the PICO framework. Studies that did not meet the inclusion criteria were excluded at this stage. Duplicate records were identified during full text screening, at which point one of the duplicate records was excluded. The 29 studies that remained were then assessed for eligibility based on the full text in accordance with the predefined PICO criteria. In the case of unclear eligibility, the study was discussed with the supervisor, and eligibility was determined based on the inclusion and exclusion criteria. A final total of 19 studies met the inclusion criteria and were included in the review.

### 1.6 Data charting process and data items

Data from all included literature was charted using a standardized data extraction form implemented in Excel prior to data extraction. The extracted items are listed below:

1. Author, year of publication.
2. Publication type.
3. Study design.
4. Sample size and participant age.
5. Type of epilepsy or epilepsy syndromes.
6. Type of KDT.
7. Duration of dietary treatment and follow‐up points after treatment initiation.
8. Seizure outcome.
9. Cognitive outcomes.
10. Method used to assess cognitive outcomes.

For all included literature, the study design was recorded to differentiate between observational studies, case series, and case reports. Study design for every included study is reported in Table 3. Similarly, publication type was recorded to distinguish conference abstracts from full‐text articles. The method of cognitive assessment was reported to distinguish between objective assessment and subjective reporting. All recordings mentioned above are presented in detail in Table 3.

**TABLE 3:** Study Characteristics and Findings of the Included Literature.

Author, year	Publication type	Study design	Sample size/age	Epilepsy type/etiology	Diet	Duration/follow up	Seizure outcomes	Cognitive outcomes	Domains	Cognitive assessment method
Sirven et al., 1999^16^	Article	Prospective study	*n* = 11; 19–45 y; mean 32.2 y	DRE; *n* = 6 cryptogenic PE; *n* = 5 SGE	KD	8 mo; first time 2 weeks after initiation, then q1mo	*n* = 3 90% seizure reduction; *n* = 3 50–80% seizure reduction; *n* = 1 < 50% seizure reduction	*n* = 2↑ concentration; *n* = 7 ↑cognition and mood; *n* = 1 no seizure improvement; continued for cognitive ↑	Concentration; cognition	Baseline assessment by neuropsychologist; Self‐report at follow‐up (subjective)
Remahl et al., 2008^17^	Article	Prospective study	*n* = 23; 1.3–15.8 y; mean 6.5; *n* = 2 adolescents (13 y & 15 y)	DRE; *n* = 6 generalized epilepsy; *n* = 6 partial epilepsy	KD	3 mo; 3 mo	*n* = 2 adolescents = >50% seizure reduction	*n* = 1 ↔; *n* = 1 ↑	QoL = Consciousness, awareness, ability to maintain contact, interest in immediate surroundings, ability to convey a message	Parental reports (subjective)
Carrette et al., 2008^18^	Article	Prospective study	*n* = 8; 31–55 y; mean 41.8 y	DRE	MAD	6 mo (*n* = 3); follow‐up q1mo	Mean seizure reduction for patients completing 6 mo (*n* = 3) = 42.2% (range: 25–60%); mean seizure reduction for patients completing more than 3 mo (*n* = 4) = 20%	*n* = 6 ↑ concentration; *n* = 3 completing 6 mo ↑ concentration; 86% reported improved cognition	Concentration; cognition	Self‐report (subjective)
Lambrechts et al., 2012^19^	Article	Prospective study	*n* = 15; 6–18 y; mean 11.9; *n* = 11 adolescents (10–18 y)	DRE	KD	2–>14 mo; outcome follow‐up 6 mo	*n* = 3 adolescents >50% seizure reduction; *n* = 5 adolescents <50% seizure reduction	*n* = 5 stayed on diet for cognitive ↑; cognitive tests showed ↑ (1.6–8.7%) but no test showed statistical significance; one test ↓ (7.7%)	Alertness, global cognition, attention, VMI	Dutch version of the Peabody Picture Vocabulary test (global cognitive level), the FePsy neuropsychological test battery (attention), and Beery's Visual‐Motor Integration (VMI) test (psychomotor functions); Self report (objective & subjective)
Schoeler et al., 2014^20^	Article	Audit/observational	*n* = 23;16–65 y; mean 30.8 y	DRE; 2 JME, 7 structural/metabolic, 1 r 17, 13 unclassified	KD; MAD	3 mo–10 y; follow‐up 3 mo, then q6mo	9/23 (39%) ≥50% seizure reduction; 5/23 (22%) less pronounced or inconsistent seizure reduction; 4/23 (17%) no change; 1/23 (4%) increased seizure frequency; 14/23 (61%) less severe/shorter seizures	15/23 (65%) more alert; 7/23 (30%) increased concentration	Alertness; concentration	Unprompted reporting (subjective)
Roehl et al., 2019^21^	Article	Retrospective study	*n* = 55, 17–70 y, mean 38 y	DRE; majority focal epilepsy	Modified KD (MKD‐15 & MKD‐50)	NR; follow up aprox. 3 mo (range 2–7)	33/55 (60%) achieved ≥50% seizure reduction; 42/55 (76%) reported improvement in seizure severity.	87% (*n* = 48) QoL ↑	mood, alertness, memory, attention, sleep	Self‐reported QoL (subjective)
Gordon et al., 2020^22^	Article	Qualitative study, Cross‐sectional on‐line survey + physical work‐shop (patients, families, carers, healthcare professionals)	*n* = 42 PFC responses, ~48% adults, (adult subgroup for cognitive outcomes NR) diet initiated mainly during childhood/adolescence (8–17 y)	DRE; Ring chromosome 20 syndrome	KD	Minimum 3 mo; ~20% maintained >2 years	30% (6) significant improvement; 15% (3) small improvement; 40% (8) no change; 10% (2) small worsening, 5% (1) significant worsening	30% (6) significant ↑; 5% (1) small ↑; 55% (11) ↔; 10% (2) small ↓; 0% (0) significant ↓	Cognition, alertness	Parent/carergiver proxy report (web‐based PFC survey) (subjective)
Green et al., 2020^23^	Article	Prospective study (single center)	*n* = 42 initiated (*n* = 40 analyzed for seizures); mean age 36.8 yrs	focal & generalized (implied DRE)	Modified Ketogenic Diet (MKD)	12 months; follow‐up at at 3, 6, 9, 12 mo	60% reported improvement; 38% ≥50% seizure reduction; 13% seizure freedom (rare sustained); 30% reported worsening	31% ↑ alertness; 10% ↑ mood; some ↓ mood ↓ 5% memory	Alertness, mood, memory	Self‐reported (subjective)
Samões et al., 2024^24^	Article	Prospective study	*n* = 39, 18–65 y, 40 completed 3 mo	DRE; focal, generalized, structural, genetic epilepsy	MAD	3 mo; follow‐up at 1 & 3 mo	At 3 mo, 51% (20/39) were responders with a mean seizure reduction of 54% (range 17–100%), including 26% (10/39) achieving ≥50% reduction (AUC = 0.683, p = 0.048) at 3 mo	Subjective cognitive ↑ in 54% (21/39) at 3 mo (AUC = 0.751, p < 0.01) at 3 mo	Awareness, behavior, mood	Semi‐structured qualitative interview patient or caregiver (subjective)
Diao et al., 2025^25^	Article	Retrospective study (single center)	*n* = 7; median 32 y, (IQR 25–39); 4 on KD	Cryptogenic NORSE	KD	Median 76.5 days; follow‐up 3 mo post‐discharge	EEG: seizure waves disappeared after 3 days of KD in some; overall residual seizures controlled; super‐refractory in acute phase	Residual cognitive and memory impairment in all surviving patients; KD did not reverse deficits	Awareness; behavior; mood; alertness; memory	Modified Rankin Scale (MRS) (subjective)
Haridas et al., 2025^26^	Article	Retrospective study (single‐center case series)	Total *n* = 9; *n* = 4 adolescents (11–15 y)	DRE; Rett syndrome; focal & generalized	KD	2–84 mo (mean 29.4); follow‐up 6 & 12 mo	6/9 (67%) ≥50% seizure reduction at 6 months; 4/9 (44%) at 12 months	2/4 adolescents (11–15 Y) ↑; none in non‐responders	alertness ↑, interactivity ↑, and activity level ↑	Parental reports (subjective)
Samões et al., 2025^27^	Article	Prospective study (Single center)	*n* = 80,16–68 y; mean 29 y	DRE; focal, generalized, structural, genetic, infectious, unknown	MAD, occasional addition of formula‐based KD/MCT	Median 122 days (range 3–2264 days), follow‐up 3 mo	12/51 (24%) achieved ≥50% seizure reduction at 3 months; qualitative improvements in seizure duration, postictal period, and falls.	Subjective improvements	Awareness, behavior, mood, cognition	Semistructured interviews; seizure calendars (subjective)
Obara & Ono, 2022^28^	Article	Case report	*n* = 1; 21 y	DRE; FIRES	KD	KD initiated 27 months post‐onset; follow‐up 3, 9, 16 mo	75% seizure reduction within 3 months	Mild improvement overall; IQ ↑ (52 → 67); WMS‐R < 50 → ↑ in some domains	IQ ↑ (52 → 67); reasoning ↑ (perceptual, verbal); attention ↑; visual memory ↑; general memory ↔; verbal memory ↔; processing speed ↔; working memory ↔; delayed recall ↔	WAIS‐IV, WMS‐R (objective)
Lapp et al., 2012^29^	Conference abstract	Retrospective study	*n* = 26; >18 y	DRE; JME, LGS	KD (*n* = 7); MAD (*n* = 19)	2 weeks–54 mo; NR	11 (42%) improved seizure control; 6 (23%) reduction in seizure severity, 8 (31%) no change; 1 (3.8%) worsening; 6 (23%) not on diet long enough to know effectiveness	*n* = 9 (35%) ↑ alertness	Alertness	Self‐report, patients notice (Subjective)
Grippo et al., 2017^30^	Conference abstract	Retrospective study (case series)	*n* = 11, 18 mo–18 y 10 mo	DRE	KD	3–31 mo; response at 6 mo	≥50% seizure reduction in 7/11 (64%); seizure freedom in 3/11 (27%)	All patients ↑	Cognitive skills, life quality	Parental reports/clinical observation (assumption as SF was from parents) (subjective)
Falco‐Walter et al., 2017^31^	Conference abstract	Retrospective study	*n* = 17, 21–59 y, mean 34 y; *n* = 13 followed MAD	Medically and surgically refractory epilepsy	MAD (*n* = 13), LGID (*n* = 4)	NR; follow‐up 3 months	Among patients achieving >50% seizure reduction, 67% (6/9) were on MAD, 71% (12/17) reported milder seizures	QoL ↑ in 82% (14/17)	Alertness, mood	Self‐reports (subjective)
Nieto & Londono, 2019^32^	Conference abstract	Case report	*n* = 3, 13 y–14 y	DRE; 2 genetic epilepsies (STXBP1, Doose), 1 idiopathic focal epilepsy	KD	NR	~60% seizure reduction in 2/3 cases, with qualitative seizure improvement in the third case.	2 patients↑, 1 NR	Cognitive performance, cognitive status	Clinical observation (subjective)
Samões et al., 2019^33^	Conference abstract	Prospective study (single‐center)	*n* = 16; mean age 32.4 y (SD 13.3)	Structural, focal, epileptic encephalopathies	MAD	Up to 6 mo, follow‐up 1, 3 and 6 mo	≥50% seizure reduction in 40% (2/5) at 3 months and 50% (1/2) at 6 months, with additional patients showing <50% reduction.	↑ in most patients	Cognition and behavior	Informal caregiver reports (subjective)
Volpi et al., 2024^34^	Conference abstract	Retrospective cohort	*n* = 27, 18–62 y, mean 33 y	DRE	KD, mild calorie restriction	Mean 9 mo; assessments at baseline, 1, 3, 6, 12 mo	KD effective in 16/27 (59.3%) with >50% seizure reduction	Mixed effects (improvement + initial transient impairments)	Alertness, social skills, disability; (transient irritability, short. term memory, concentration ↓)	NR (subjective)

*Note*: Values are reported for the full cohort as described in the original studies, unless otherwise specified. Subgroup samples, e.g., dietary therapy only or age‐specific groups, are reported where applicable.

Abbreviations: ↑, improvement; ↓, worsening; ↔, no change; BL, baseline; Cryptogenic PE, Cryptogenic partial epilepsy; DRE, drug‐resistant epilepsy; EEG, electroencephalogram; FePsy, The Iron Psyche neuropsychological test battery; FIRES, febrile infection‐related epilepsy syndrome; IQR, interquartile range; JME, juvenile myoclonic epilepsy; KD, ketogenic diet; LGID, low glycemic index diet; LGS, Lennox Gastaut syndrome; MAD, modified Atkins diet; MCT, medium‐chain triglycerides; MKD, modified ketogenic diet; mo, months; *n*, number of participants; NORSE, new‐onset refractory status epilepticus; NR, not reported; PFC, Patient/family/caregiver; q1mo, every month; q6mo, every 6 months; QoL, quality of life; SD, standard deviation; VMI, visual‐motor integration; WAIS‐IV, Wechsler Adult Intelligence Scale‐IV; WMS‐R, Wechsler Memory Scale‐Revised; y, years.

### 1.7 Critical appraisal of individual sources of evidence

The included literature did not go through a critical appraisal process as the aim of this scoping review was to map all available evidence.

## 2 RESULTS

### 2.1 Synthesis of results

Results include descriptive tables presenting the key findings and a narrative summary describing patterns in the literature.

### 2.2 Selection of sources of evidence

The study selection process is presented in Figure 1. The search identified a large literature base, which the inclusion criteria narrowed to 19 studies. Both peer‐reviewed articles and conference abstracts were included to provide a comprehensive overview of available evidence.

**FIGURE 1:**
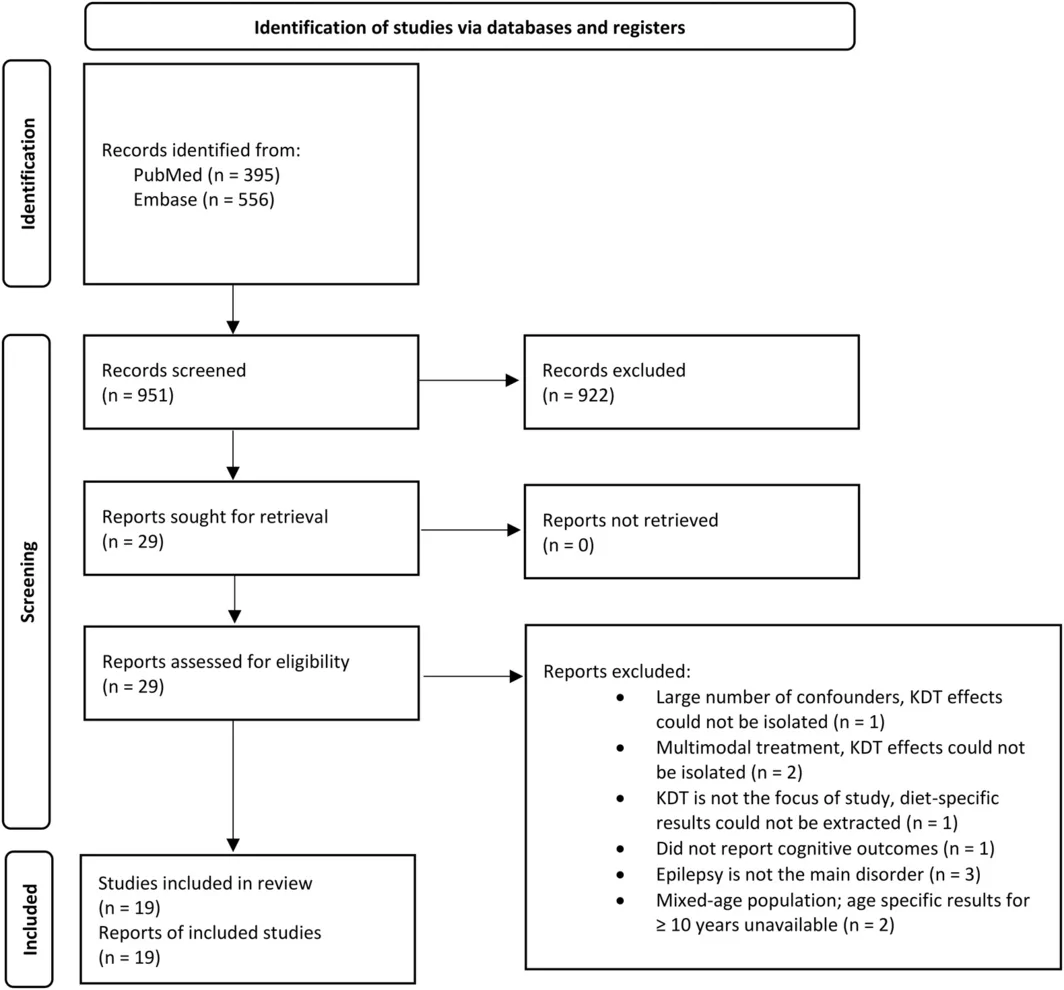
PRISMA Flow Diagram of Study Selection through Database Searches.

### 2.3 Synthesis of results

The results show a pattern of improvements in cognitive functioning following KDT. This pattern was commonly linked with favorable seizure outcomes, although that linkage was inconsistent. Table 3 summarizes the data extracted according to the predefined items.

### 2.4 Characteristics of sources of evidence

Included studies used heterogeneous designs, the most common of which was prospective cohorts (*n* = 8), followed by retrospective designs (*n* = 7), case reports (*n* = 2), cross‐sectional (*n* = 1), and audit (*n* = 1) designs. Sample sizes ranged from one to 80 participants, with ages from 15 months to 68 years. Most studies included DRE across focal, generalized, genetic, structural, infectious, idiopathic focal, and refractory status epilepticus populations.

### 2.5 Dietary treatment

Most studies used KD (*n* = 9), followed by MAD (*n* = 4), or a combination of both (*n* = 2). Some studies included variations such as low‐glycaemic‐index diet, modified KD, formula‐based KD, or MCT supplementation. Treatment duration ranged from 2 weeks to approximately 10 years, while reported follow‐up time points ranged from 2 weeks to 16 months after treatment initiation.

### 2.6 Seizure outcomes

All included studies (*n* = 19) reported seizure improvement following dietary therapy, most commonly defined as ≥50% seizure reduction. Three studies reported seizure aggravation or worsening of seizures in some patients^22^, ^23^, ^29^. Two studies described seizure freedom, where one reported seizure freedom in three patients on KD,^30^ whereas the other reported 13% of participants experiencing seizure freedom, with one sustaining it at 12 months.^23^ Additional qualitative improvements were reported, including milder seizures, shorter seizure durations, and improvements in the postictal period. Two studies observed no change in seizure frequency.^22^, ^29^ Where data were available, most improvements occurred within the first follow‐up after treatment initiation, ranging from 1 to 7 months.

### 2.7 Cognitive outcomes

The definition of cognitive outcomes varied considerably across studies and included constructs extending beyond the strict neuropsychological definition of cognition. In addition to memory and other cognitive abilities, studies assessed alertness, awareness, mood, behavior, and sleep‐related outcomes. Less commonly reported domains included global cognition, psychomotor functioning, attention/sustained attention, concentration, consciousness, environmental awareness, communication ability, interactivity, activity level, social skills, and disability. One case report used IQ as a broader measure of cognitive functioning (Table 3).

Most studies (*n* = 18) reported cognitive improvements following dietary therapy, typically subjectively. For example, Samões et al.^24^ reported subjective cognitive improvements in 54% of patients, while Obara & Ono^28^ reported an increase in IQ from 52 to 67 after 16 months of dietary treatment in a 21‐year‐old patient with febrile infection‐related epilepsy syndrome (FIRES). Lambrechts et al. (2012) reported a positive trend toward improvement across all but one cognitive test, although none of the results reached statistical significance. Four studies reported residual impairment, worsening, no cognitive improvement/change, or mixed effects.^19^, ^26^, ^29^, ^34^

### 2.8 Methods of assessment

Apart from two studies, cognition was assessed subjectively through patient, clinician, carer, or parent reports. Other methods included semi‐structured interviews, quality‐of‐life scales, and the Modified Rankin Scale. Obara & Ono^28^ used standardized objective measures including the Wechsler Adult Intelligence Scale‐IV (WAIS‐IV) and Wechsler Memory Scale‐Revised (WMS‐R). Similarly, Lambrechts et al. (2012) applied objective measures using the Dutch version of the Peabody Picture Vocabulary test (PPVT), The Iron Psyche (FePsy) neuropsychological test battery, and Beery's Visual‐Motor Integration test (Beery VMI). The study also reported subjective parental reports.^19^

### 2.9 Relationship between seizure and cognitive outcome

Where reported, improvements in cognition were often aligned with seizure reduction (*n* = 16). However, this relationship was not consistent across all studies. For example, one study reported cognitive improvements in all patients, but seizure reduction ≥50% in 64% of the patients.^30^ In contrast, one study reported 5 out of 11 adolescent participants continuing the dietary treatment for its cognitive improvements, despite experiencing <50% reduction in seizure frequency.^19^

## 3 DISCUSSION

### 3.1 Summary of results

This scoping review mapped 19 studies on KDT in adolescents and adults with epilepsy, most involving DRE. Seizure outcomes were generally favorable, and most studies reported cognitive improvements. Cognitive outcomes were primarily measured by subjective reports; only two studies used standardized neuropsychological testing.

### 3.2 Seizure control and cognitive outcomes

Cognitive improvements often aligned with seizure reduction, but the association was inconsistent. This suggests that reduced seizure burden may contribute to improved cognition, while baseline cognitive functioning, disease severity, comorbidities, and treatment history may also shape outcomes. These findings warrant further investigation into the potential relationship between seizure reduction and cognitive outcomes following dietary therapy.

In severe refractory cases,^25^, ^28^ KD was used alongside multiple ASMs and anesthetic treatment, limiting the attribution of effects to diet alone. Although seizure reductions were reported, residual cognitive impairment persisted. KD may have an indirect protective role if it reduces reliance on high‐dose anticonvulsants, but evidence is limited by very small samples, concurrent treatments, and clinical variability.

### 3.3 Cognitive assessment and outcome measurement

Overall, current evidence does not support firm conclusions about objective cognitive improvement after KDT in adolescents and adults with epilepsy. Alertness was most frequently assessed, but it captures only one aspect of cognition and does not replace domain‐specific cognitive testing. Objective measures such as WAIS‐IV and WMS‐R, used in one case report, and PPVT, FePsy, Beery VMI, used in a prospective study, provide more detailed domain‐specific information. Interpretation is limited by the narrow range of cognitive domains assessed and the reliance on subjective reports in the few studies that did report cognitive outcomes.

### 3.4 Population heterogeneity and variability in treatment response

Included populations varied substantially in epilepsy type, disease severity, baseline cognition, age, and concurrent treatments. Genetic epilepsies and severe refractory conditions may follow distinct cognitive trajectories, making treatment effects difficult to isolate.

### 3.5 Limitations and strengths

The main limitations include small samples, non‐randomized designs, a lack of control groups, a reliance on subjective cognitive outcomes, and substantial clinical heterogeneity. Changes in ASMs, VNS use, and comorbidities further limit causal interpretation. Although the inclusion of several conference abstracts reduces methodological transparency, they were retained to comprehensively map the available findings. Strengths include the use of PRISMA‐ScR guidance, transparent eligibility criteria, and the inclusion of both peer‐reviewed and preliminary evidence to map a limited field comprehensively.

### 3.6 Evidence gaps and future research

There is a clear lack of objective evidence regarding cognitive outcomes following dietary therapies in adolescent and adult epilepsy populations. Future studies should use standardized cognitive assessments, prospective controlled designs, larger samples, and longer follow‐up periods.

## 4 CONCLUSION

Overall, the research on cognitive outcomes based on randomized controlled studies following KDT in adolescents and adults with epilepsy is limited. Most studies reported favorable seizure and cognitive outcomes, although the evidence was predominantly subjective and based on heterogeneous designs. These findings highlight the importance of further research to advance our understanding of the effects of dietary therapies on cognitive functioning in individuals with epilepsy. Future research should include larger prospective controlled studies, standardized cognitive assessments, and longer follow‐up periods.

## AUTHOR CONTRIBUTIONS

TI conducted the literature search and wrote the initial draft. TI and ASR designed the review strategy. All authors contributed to the interpretation of findings and critical revision of the manuscript. ASR supervised the project. All authors approved the final version.

## FUNDING INFORMATION

This research received no external funding.

## CONFLICT OF INTEREST STATEMENT

None of the authors have any conflicts of interest to disclose.

## ETHICAL STATEMENT

We confirm that we have read the Journal's position on issues involved in ethical publication and affirm that this report is consistent with those guidelines.

## Data Availability

Data sharing not applicable to this article as no datasets were generated or analysed during the current study.
